# Experimental Study on the Filtration of Seawater Bentonite Slurry Under the Cutting Influence of Shield Cutterhead

**DOI:** 10.3390/ma18174025

**Published:** 2025-08-28

**Authors:** Deming Wang, Zhipeng Li, Qingsong Zhang, Lianzhen Zhang, Yang Gao, Hongzhen Dong, Yirui Li, Yueyue Wu, Yongqi Dai

**Affiliations:** 1 School of Transportation and Civil Engineering, Shandong Jiaotong University, Jinan 250357, China; wangdeming@sdjtu.edu.cn (D.W.); donghongzhen630@outlook.com (H.D.); lyr23107019@stu.sdjtu.edu.cn (Y.L.); 204157@sdjtu.edu.cn (Y.W.); daiyongqisdjtxy@163.com (Y.D.); 2 School of Civil Engineering, Shandong University, Jinan 250100, China; zhangqingsong@sdu.edu.cn; 3 College of Pipeline and Civil Engineering, China University of Petroleum, Qingdao 266580, China; zhanglianzhen@upc.edu.cn; 4 School of Safety Engineering and Emergency Management, Shijiazhuang Tiedao University, Shijiazhuang 050043, China; gaoyang_sjz@163.com

**Keywords:** slurry shield tunneling, bentonite slurry materials, cutterhead cutting, effective support force conversion rate, influencing factors

## Abstract

Slurry shields maintain excavation face stability by forming a sealing filter cake through pressurized slurry filtration, though cutterhead rotation inevitably compromises this integrity. This study investigates seawater-based slurry filtration behavior under cutterhead disturbance using model testing, utilizing the effective support force conversion rate to quantify the filter cake formation efficiency. Quantitative analysis evaluated key slurry constituents—bentonite, carboxymethyl cellulose (CMC), and fine sand (content/particle size)—and operational parameters including cutterhead rotation speed, advance rate, and slurry pressure. Results demonstrate enhanced conversion rate and stability with increased bentonite, CMC, and fine sand content; reduced fine sand particle size; elevated slurry pressure; and decreased cutterhead speed/advance rate. Nonlinear relationships exist between bentonite content and fine sand particle size, on the one hand, and the mean conversion rate and its fluctuation range, on the other. Stratum permeability and slurry pressure exhibit nonlinear effects on fluctuation range but linear relationships with mean value, indicating marginal impacts on support force magnitude and operational stability. Sensitivity analysis confirms bentonite as the dominant influencing factor, followed by cutterhead speed and CMC.

## 1. Introduction

Slurry shields rely on a pressurized slurry to form a filter cake at the excavation face, balancing water and soil pressures. This technique allows tunneling through strata with poor self-stability, high groundwater pressure, and high permeability. It is now widely used in constructing tunnels across rivers and seas. Water-rich rock fracture is a typical poor geology condition often encountered in tunnel and underground construction engineering [1]. During subsea tunnel construction, the bentonite slurry used in slurry shield tunneling can lose rheological properties due to seawater contamination, which negatively affects the stability of the excavation face [2], especially when the shield crosses subsea fault zones [3]. Therefore, studying the filtration process of slurry in fractured and highly permeable strata, along with the mechanism of filter cake formation during seawater intrusion, is crucial to maintaining the stability of the excavation face in difficult geological conditions, such as subsea faults and fractured zones.

Most studies on the filtration mechanism of slurry at the excavation face are conducted using one-dimensional slurry filtration devices. These devices are usually cylindrical, with stratum materials placed inside. Under air pressure, the slurry forms a filter cake on the surface of the stratum material, and the filtrate is discharged from the other end of the device. Many researchers have used such devices to conduct extensive studies on the filtration behavior of slurry under static cutterhead conditions, examining factors such as slurry materials and stratum conditions. These studies have shown that slurry density, viscosity, clay content, and particle size in the slurry significantly influence the quality of the filter cake [4,5,6,7,8].

During slurry balance shield tunneling, the filter cake at the excavation face undergoes a repeated cycle of “destruction–formation–destruction”. Experiments based on one-dimensional slurry filtration devices cannot accurately reflect the working conditions of slurry during tunneling. Some researchers have modified these devices by adding cutting tools and power systems to study how the filter cake forms under dynamic conditions. Results showed that the slurry filtration rate during cutterhead rotation was much higher than during static conditions. The slurry filtration loss was found to be directly proportional to the cutterhead rotation speed and inversely proportional to the slurry pressure, while the excess pore water pressure in the soil was inversely proportional to the distance from the excavation face [9,10,11]. Compared to slurry filtration tests, shield tunneling model tests offer a more complete simulation of the excavation process. Some researchers have used these models to study how tunneling affects surface settlement, soil stress, and failure modes at the excavation face [12,13,14,15,16,17,18].

Some studies have developed theoretical models for filter cake formation that take into account the cutting action of the cutterhead. These models examine the effects of cutterhead rotation speed and slurry pressure on the thickness, elastic modulus, formation time, and effective stress of the filter cake. The models indicate that cutterhead excavation periodically disrupts the pressure transmission mechanism, and different combinations of cutterhead rotation speed and penetration depth can lead to variations in slurry pressure transmission [19,20,21,22,23,24]. These studies are of great significance for setting tunneling parameters, such as cutterhead rotation speed, slurry pressure, and advance speed, as well as for maintaining the stability of the excavation face during slurry shield tunneling.

Bai Yang [9] primarily investigated the mechanisms of slurry infiltration and filter cake formation at the excavation face of slurry shields, as well as the stability of the excavation face under “cutter–soil” interaction. This study revealed how the cutting effects of shield cutterheads and tools influence filter cake characteristics, formation mechanisms, and excavation face stability. Mao Jiahua [25] employed theoretical analysis, laboratory tests, and numerical simulations to study the patterns and mechanisms of slurry infiltration and filter cake formation in sandy strata, exploring the slurry support mechanism at the excavation face under cutterhead and tool action. However, these studies primarily focused on the impact of factors like cutterhead opening ratio, cutter arrangement, and cutterhead rotational speed on filter cake formation and excavation face stability, with limited attention to the influence of slurry properties. Furthermore, no existing research has quantitatively analyzed the effects of bentonite, additives, slurry pressure, cutterhead rotational speed, and other factors on excavation face support effectiveness under cutter cutting conditions. In contrast, studies represented by Su Qin et al. [26] investigated slurry infiltration in sandy and similar strata. These studies typically utilized slurries prepared with fresh water, neglecting the effects of ions in groundwater (such as flocculation and sedimentation) on bentonite under subsea conditions, and did not consider the influence of cutter cutting on filter cake formation.

Based on a slurry shield tunneling model testing device, this study investigates the filtration behavior of seawater-based slurry at the excavation face under the cutting action of the cutterhead. By analyzing the changes in pore water pressure in the stratum during slurry filtration, the effects of slurry materials—such as bentonite, sodium carboxymethyl cellulose, and fine sand—along with cutterhead rotation speed, advance speed, slurry pressure, and stratum conditions on slurry filtration are studied. The role of these factors in maintaining the stability of the excavation face is also analyzed.

## 2. Experimental Apparatus and Procedures

**Figure 1:**
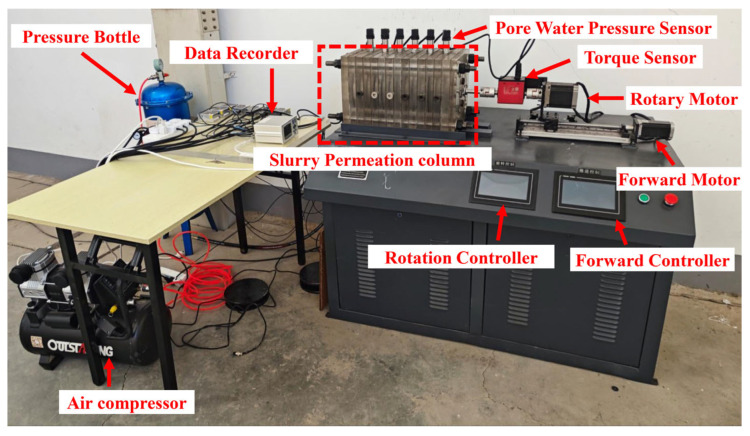
Slurry shield tunneling model testing device [27].

To investigate the filtration characteristics of seawater-based slurry under cutterhead disturbance during cutter excavation, a slurry permeation test apparatus was developed. This apparatus enables permeation tests under both cutter excavation and static cutter conditions, with preliminary experiments under non-excavating conditions having been previously conducted [27]. The experimental apparatus (Figure 1) constitutes a modified one-dimensional slurry filtration and cake formation system, comprising a slurry filtration column, cutting mechanism, data acquisition system, and pressure supply unit. This configuration simulates excavation face slurry filtration processes under cutterhead cutting action. During testing, cutterhead rotation speed and advance rate are dynamically controlled through torque and thrust adjustment, while real-time monitoring captures slurry pressure, stratum pore water pressure, and drive shaft torque.

**Figure 2:**
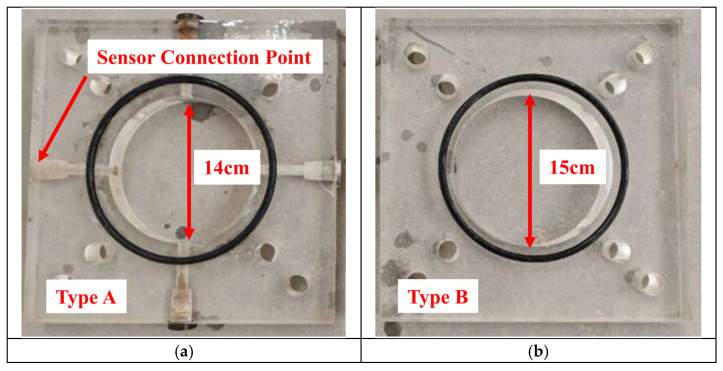
Two types of PMMA plates [27]: (**a**) type A acrylic board with sensor interface (**b**) type B acrylic board.

The filtration column, which is filled with formation materials, is the main component for conducting the filtration test. It is assembled using PMMA plates with different diameters, creating internal cavities, as shown in Figure 2. The alternating installation of two types of PMMA plates forms a zigzag-shaped internal space within the filtration column. This design prevents the compression of the stratum under pressure and avoids slurry filtration through gaps between the stratum and the container wall [28]. Type A PMMA plates have interfaces on the side walls for sensor connections, while unused interfaces are sealed with bolts.

The cutterhead is connected to the top cover of the filtration column. It is welded to the drive shaft, which is connected to the top cover through a rotating seal. This sealing mechanism allows the drive shaft to rotate and extend while maintaining the seal of the top cover. The other side of the drive shaft is connected to the power system via coupling.

**Figure 3:**
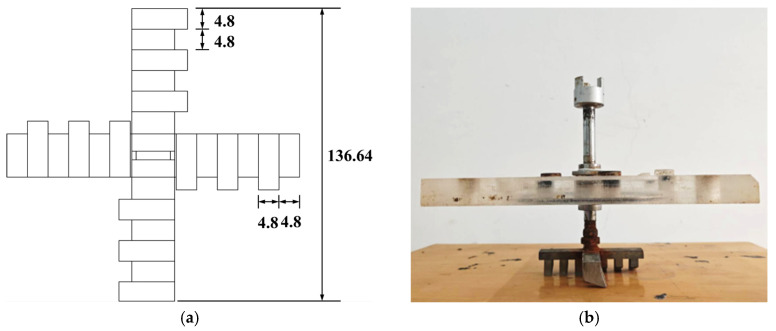
Schematic diagram of the cutterhead: (**a**) Cutterhead dimensions (mm) and (**b**) cutterhead and top cover plate.

Generally, scraper cutters are employed to excavate stiff, compacted soils, while disk cutters are used for rock fragmentation [29]. In sandy strata, slurry shield tunneling predominantly utilizes scraper cutters; therefore, scraper cutters were selected for the cutting tools in this experimental setup [10]. The cutterhead features a symmetrical four-arm design, with three cutters mounted on each arm and a central scraper blade installed at its center. The cutterhead configuration and installation methodology are illustrated in Figure 3.

The cutting system consists of a thrust motor, a transmission mechanism, and a rotary motor. The transmission mechanism converts the rotational motion of the thrust motor into horizontal movement, allowing the rotary motor to drive the cutterhead to cut the excavation face. The data acquisition system is used to collect data such as pore water pressure, torque, and the weight of slurry filtrate during the experiment. The pressure supply system consists of an air compressor, a pressure regulator, and a pressure tank, which provide power for the slurry filtration process.

**Figure 4:**
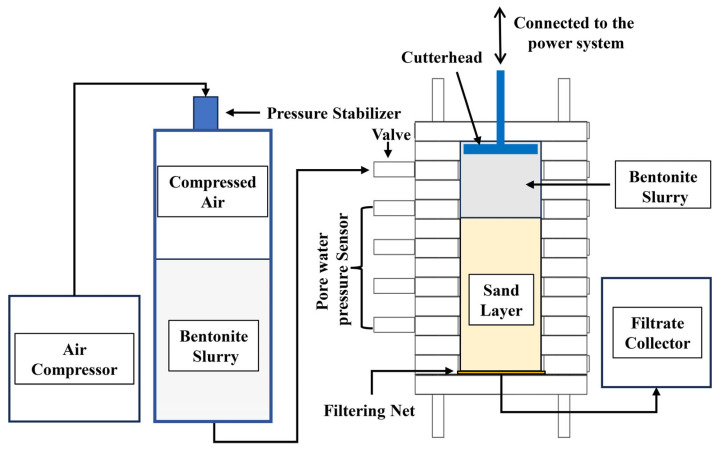
Schematic diagram of the experimental setup [27].

A schematic diagram of the experimental setup is shown in Figure 4. The device uses compressed air to drive the slurry filtration process. The slurry is placed in a pressurized steel tank and is pushed by compressed air at a fixed pressure through a pipeline into the filtration column. The slurry is filtered by the sand layer material inside the filtration column, and the filtrate is collected in a container. During slurry filtration, the cutterhead cuts the surface of the sand layer, driven by two motors. Pore water pressure sensors mounted on the side wall of the filtration column record the changes in slurry pressure and pore water pressure in the stratum throughout the experiment.

Due to the cutting action of shield cutter tools, a complete filter cake cannot form at the excavation face. This allows continuous slurry infiltration into the surrounding stratum, which leads to an increase in excess pore water pressure within the formation. The generation of pore water pressure reduces the effective stress acting on the soil mass [30,31,32].

To better illustrate the variation in effective support pressure acting on the excavation face during slurry infiltration, ω is defined as the Effective Support Pressure Conversion Rate (Equation (1)). This parameter characterizes the ability of the filter cake to transform slurry pressure into effective stress [33]. A higher Effective Support Pressure Conversion Rate corresponds to greater effective stress acting on the filter cake particles and consequently enhances excavation face stability. Therefore, analysis of the variation in this parameter enables the assessment of filter cake formation status at the excavation face during cutterhead cutting [19,34].

(1) $$\omega =\frac{P_{s}-P_{h}-P_{e}}{P_{s}-P_{e}}$$

In this equation, $P_{S}$ is slurry pressure (MPa), $P_{h}$ is hydrostatic pressure at the measurement point (MPa), and $P_{e}$ is excess pore water pressure at the measurement point (MPa).

When conducting slurry filtration tests with cutterhead cutting, a filtration test without cutterhead cutting is first performed to allow a stable filter cake to form at the excavation face. This step is taken to prevent the surface of the sand layer inside the filtration column from collapsing when the column is mounted horizontally on the test bench. Afterward, the valve is closed, the filtration column is mounted horizontally on the test bench, and the cutterhead drive shaft is connected to the power system. The cutterhead rotation speed and advance speed are set by the controller, and the test begins.

## 3. Experimental Materials and Test Design

### 3.1. Slurry Preparation

**Figure 5:**
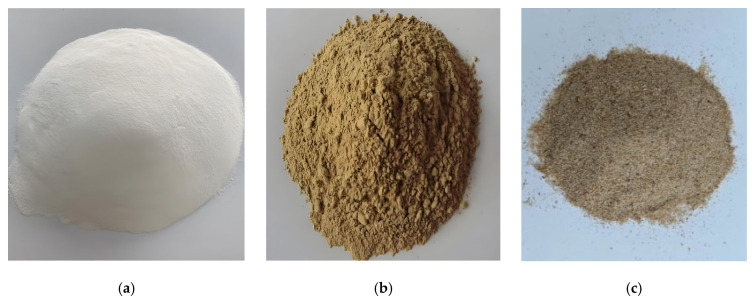
Slurry materials: (**a**) CMC; (**b**) bentonite; and (**c**) fine sand.

The materials used to prepare the slurry are shown in Figure 5, including sodium-based bentonite, CMC (Sodium Carboxymethyl Cellulose), fine sand (particle size less than 1 mm), freshwater, and artificial seawater.

Artificial seawater was prepared according to the ASTM D1141-98 [35] standard, with trace components such as potassium bromide, strontium chloride, and sodium fluoride omitted due to their very low concentrations. The proportions of the components in the artificial seawater are shown in Table 1.

When preparing the slurry, CMC is mixed evenly into the bentonite. The mixture is then gradually added to the mixing water and stirred in a mixer for 20 min. Fine sand is added and stirred for an additional 10 min. After mixing, the slurry is placed in a sealed container and allowed to stand for 24 h. Before conducting the slurry filtration test, the slurry is stirred again for 30 min.

### 3.2. Sand Layer Material

**Figure 6:**
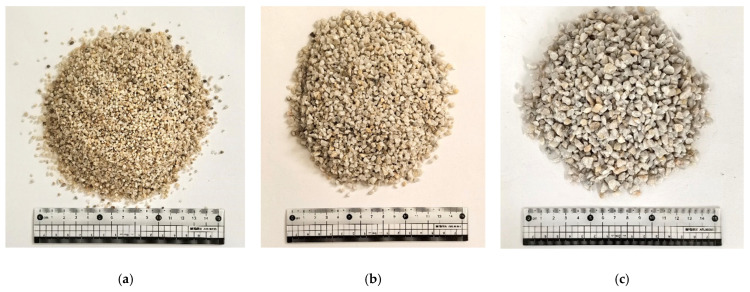
Sand layer materials: (**a**) 1–2 mm quartz sand; (**b**) 2–4 mm quartz sand; and (**c**) 4–8 mm quartz sand.

Three types of stratum materials were used in the experiment, consisting of quartz sand with particle sizes of 1–2 mm, 1–4 mm, and 4–8 mm (Figure 6). These materials can effectively simulate the characteristics of fault fracture zones and weathered troughs, which are highly fractured, have poor integrity, and exhibit strong permeability [35].

The permeability coefficients of the three materials, tested using the constant head method, are shown in Table 2.

### 3.3. Test Design

To simulate filter cake formation under cutterhead excavation conditions during subsea shield tunneling, a series of slurries was prepared by adjusting the content of bentonite, carboxymethyl cellulose (CMC), and fine sand, along with the particle size distribution of the fine sand. The slurry formulations were designed based on construction experience in highly permeable strata, including those encountered in the Foshan–Dongguan Intercity Railway’s Shiziyang Tunnel [36] and the Suai Passage project in Shantou traversing medium-coarse sand layers and highly weathered conglomeratic sandstone [37]. Under seawater salinity exposure, bentonite in the slurry undergoes flocculation and sedimentation, causing significant deterioration of key properties such as colloidal stability and viscosity. This performance degradation correlates directly with the proportion of seawater in the mixing water. When seawater constitutes 0–30% of the total mixing water, slurry properties deteriorate rapidly with increasing seawater content. When seawater content exceeds 30%, significant segregation is observed in sodium bentonite slurry, with further seawater increases exerting minimal additional impact on slurry performance [35,36,37,38,39]. To investigate seawater’s effect on slurry permeability, all test slurries were prepared with mixing water containing 30% artificial seawater. Concurrently, to examine bentonite’s role in slurry stability, additional formulations were tested, with bentonite-to-water mass ratios of 0.3, 0.35, 0.4, 0.45, and 0.6.

The experiments were conducted in strata with a particle size of 1–2 mm, a slurry pressure of 0.3 MPa, an advance speed of 10 mm/min, and a cutterhead rotation speed of 4 r/min.

Using slurry formula No. 4 from Table 3, 11 sets of experiments were conducted under different slurry pressures, cutterhead rotation speeds, advance speeds, and stratum conditions. The test arrangement is shown in Table 4. The slurry pressure, cutterhead rotation speed, and advance rate employed in the tests were determined based on operational parameters from shield tunneling at the Jintang Subsea Tunnel [40,41] and experimental configurations established in shield excavation model tests conducted by Zhang Henghui [40].

## 4. Test Results

The pore water pressure data collected during the slurry filtration process under cutterhead cutting were organized, and the effective support force conversion rate was calculated using Equation (1). The relationship between experimental time, stratum pore water pressure, and effective support force conversion rate was obtained under various conditions. Section 4.1, Section 4.2, Section 4.3, Section 4.4, Section 4.5, Section 4.6, Section 4.7 and Section 4.8 will discuss the effects of each influencing factor on stratum pore water pressure and effective support force conversion rate.

### 4.1. Bentonite Content

**Figure 7:**
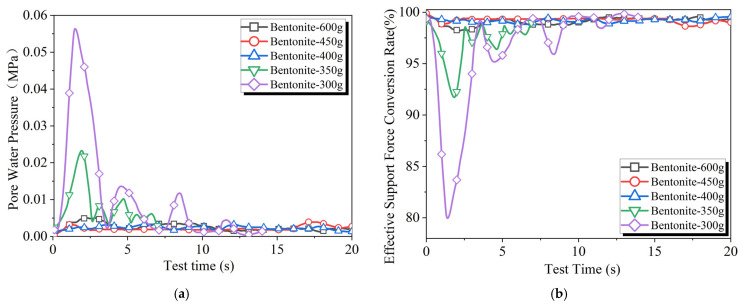
Experimental results of slurry infiltration with different amounts of bentonite: (**a**) pore water pressure and (**b**) effective support force conversion rate.

Five sets of slurries were prepared with varying amounts of bentonite for testing. The corresponding results appear in Figure 7. The results indicate significant differences in the pore water pressure curves and effective support force conversion rate curves when the bentonite content is greater than or less than 400 g. The amount of 400 g of bentonite serves as a boundary, dividing the curves into two categories. When the bentonite content exceeds 400 g, the effective support force conversion rate shows minimal fluctuations over time. Conversely, when the bentonite content is less than 400 g, the effective support force conversion rate experiences substantial fluctuations immediately after the test begins, with the level of fluctuation gradually decreasing and stabilizing as the test progresses. This phenomenon was also observed in the study by Bai Yang [42].

The reason for the two categories of effective support force conversion rate curves when varying the bentonite content is that a permeable zone forms behind the excavation face. Following the start of the experiment, under the cutting action of the tools, solid particles from the slurry accumulate within the stratum as they permeate through the excavation face, altering the stratum’s permeability. With a lower bentonite content, the range of the permeable zone formed is larger. Due to the slow advance speed of the cutterhead, the cutting action cannot rapidly disrupt the stratum that has been blocked by bentonite particles, leading to a slower rate of slurry permeation into the stratum. However, when the bentonite content is greater than 400 g, after the filter cake is damaged by the cutterhead, the slurry can quickly form a new filter cake at the excavation face, with particles in the slurry accumulating close to the excavation face, resulting in minimal changes to the stratum’s permeability.

### 4.2. CMC Content

**Figure 8:**
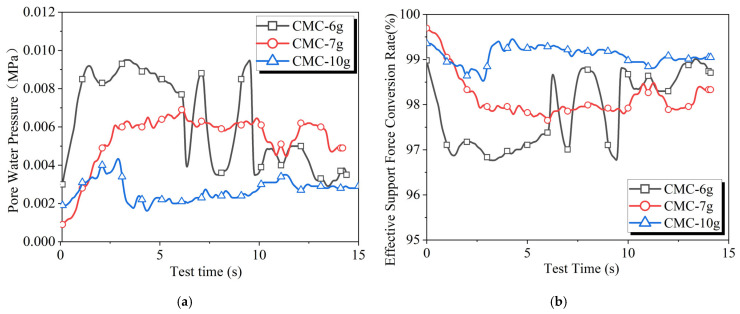
Experimental results of slurry infiltration with different amounts of CMC: (**a**) pore water pressure and (**b**) effective support force conversion rate.

As shown in Figure 8, the increase in CMC content leads to a decrease in pore water pressure and an increase in the effective support force conversion rate, while also reducing the fluctuations of both parameters. When CMC is increased from 6 g to 7 g, the fluctuations in the pore water pressure and effective support force conversion rate curves decrease. Furthermore, when the CMC content reaches 10 g, the effective support force conversion rate significantly improves, indicating that CMC has a greater impact on the stability of the effective support force conversion rate.

### 4.3. Fine Sand Content

**Figure 9:**
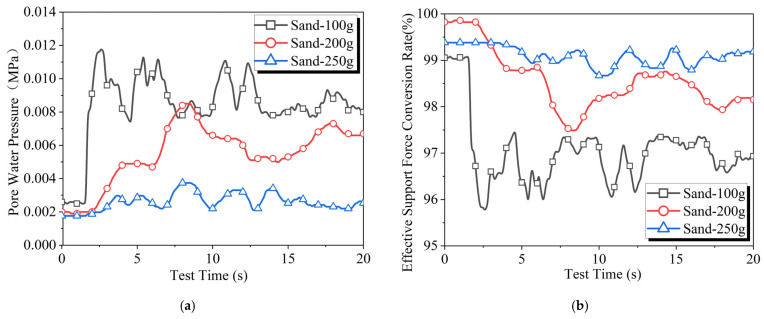
Experimental results of slurry infiltration with different amounts of fine sand: (**a**) pore water pressure and (**b**) effective support force conversion rate.

The results of the slurry filtration tests under different fine sand content conditions are shown in Figure 9. As the fine sand content increases from 100 g to 200 g, the effective support force conversion rate curve shifts upward, but the level of fluctuations does not significantly decrease. When the fine sand content is further increased to 250 g, the effective support force conversion rate curve continues to rise, and the fluctuations in the curve significantly decrease.

Comparing the test results of slurries with different fine sand contents and varying CMC contents reveals that the two components have different effects on the average level and fluctuation degree of the effective support force conversion rate during slurry filtration. CMC and fine sand represent different aspects of slurry performance, with CMC affecting the rheological properties of the slurry and fine sand influencing particle gradation. This indicates that the rheological properties of the slurry have a significant impact on the stability of the support force at the excavation face, while the particle gradation of the slurry has a greater effect on the magnitude of the support force.

### 4.4. Fine Sand Particle Size

**Figure 10:**
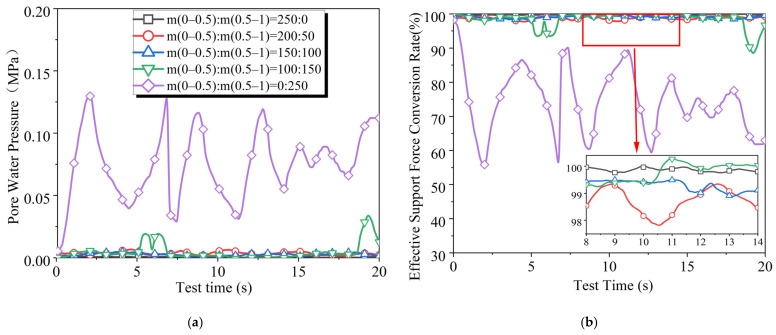
Experimental results of slurry infiltration with different amounts of fine sand size: (**a**) pore water pressure and (**b**) effective support force conversion rate.

The results of the slurry filtration tests under different fine sand particle size conditions are shown in Figure 10. When the fine sand consists entirely of particles with a size of 0.5–1 mm, the effective support force conversion rate fluctuates significantly. However, this phenomenon is markedly improved by adding small particles of size 0–0.5 mm to the fine sand. As the proportion of smaller particles continues to increase, the effective support force conversion rate increases while the degree of fluctuation decreases. This differs from the results obtained in non-cutting conditions.

The results of non-cutting slurry filtration tests conducted under the same conditions indicate that the optimal ratio for minimizing the pore water pressure generated during the formation of the filter cake is m(0–0.5):(0.5–1) = 2:3 [27]. The reason for this may be that the cutterhead’s destruction of the filter cake allows more fine particles to remain within the stratum behind the excavation face, leading to improved sealing of the stratum’s pores by the finer slurry.

### 4.5. Cutterhead Rotation Speed

**Figure 11:**
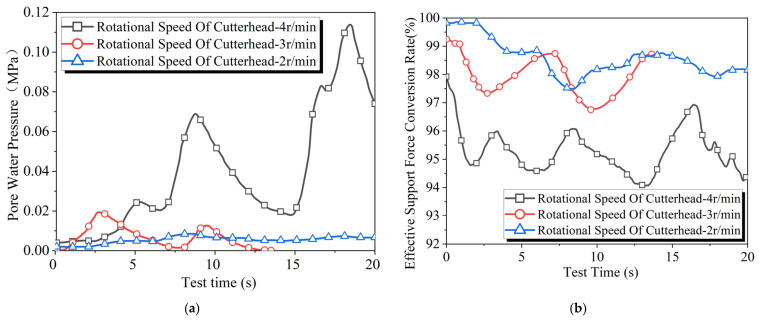
Results of slurry filtration experiments at different cutterhead rotation speeds: (**a**) pore water pressure and (**b**) effective support force conversion rate.

Figure 11 shows the test results obtained under varying cutterhead rotation speeds. The increase in cutterhead rotation speed accelerates the destruction of the filter cake, allowing more slurry to permeate through the excavation face. This leads to an increase in stratum pore water pressure and a decrease in the effective support force conversion rate. A significant change in stratum pore water pressure and effective support force conversion rate is observed when the cutterhead rotation speed increases from 3 r/min to 4 r/min. This notable change point has also been observed in the studies by Bai Yang [9] and Mao Jiahua [25], although at different positions, specifically 23 r/min and 1–2 r/min, respectively. The position of this change point may depend on factors such as slurry performance, stratum permeability, cutterhead characteristics, and slurry pressure.

### 4.6. Advanced Speed

**Figure 12:**
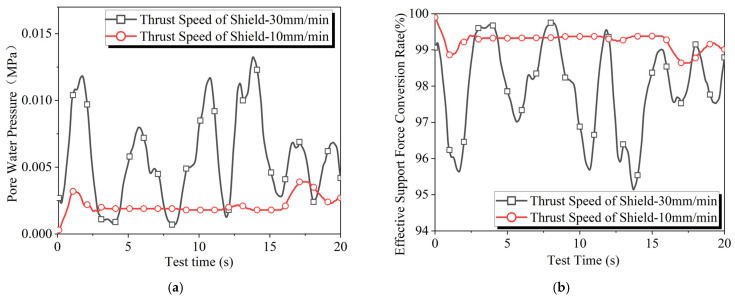
Results of slurry filtration experiments at different advance speed: (**a**) pore water pressure and (**b**) effective support force conversion rate.

Test results at different advance speed are shown in Figure 12. The increase in advance speed significantly enhances the fluctuations in the effective support force conversion rate curve, while having minimal impact on the average value of the curve. This is because the increased advance speed of the shield allows the cutter to cut deeper, which exacerbates the damage to the permeable zone behind the excavation face. This weakens the slurry particles’ ability to block the stratum, necessitating more slurry to permeate behind the excavation face to rebuild the permeable zone, thereby increasing the fluctuations in the effective support force conversion rate. Higher slurry pressure helps form a denser filter cake and accelerates the filter cake formation process [26,27]. However, it also enlarges the range of pore water pressure effects caused by slurry infiltration into the stratum [43], which increases the disturbance experienced by the stratum.

### 4.7. Slurry Pressure

**Figure 13:**
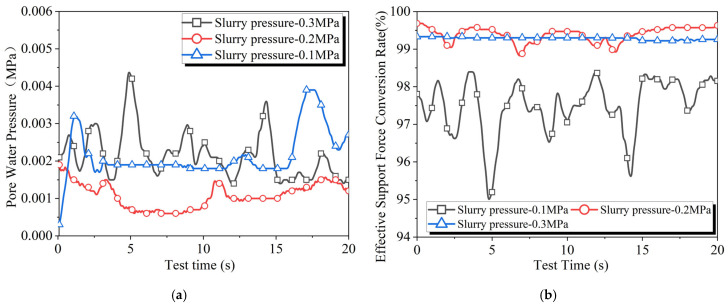
Results of slurry filtration experiments at different slurry pressure: (**a**) pore water pressure and (**b**) effective support force conversion rate.

In Figure 13a, the pore water pressure curves during the slurry filtration process under different pressures intersect with each other. In contrast, in Figure 13b, the effective support force conversion rate curve at a pressure of 0.1 MPa does not intersect with the other curves. This indicates that increasing the slurry pressure does not significantly raise the stratum pore water pressure; rather, it increases the ratio of effective stress exerted on the filter cake to the slurry pressure. This suggests that the impact of slurry pressure on the actual support force experienced by the excavation face is twofold: increasing the slurry pressure can raise both the upper limit of effective support force and its conversion ratio. It also indicates that, compared to pore water pressure, the effective support force conversion rate is a clearer indicator of the actual supporting effect of the slurry pressure on the excavation face.

### 4.8. Stratum Permeability

**Figure 14:**
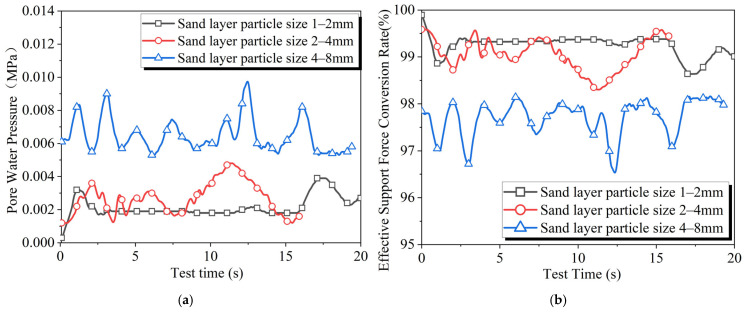
Results of slurry filtration experiments at different stratum permeability: (**a**) pore water pressure and (**b**) effective support force conversion rate.

As shown in Figure 14, as the stratum particle size increases, the stratum pore water pressure rises while the effective support force conversion rate decreases, and the level of fluctuations also increases.

**Figure 15:**
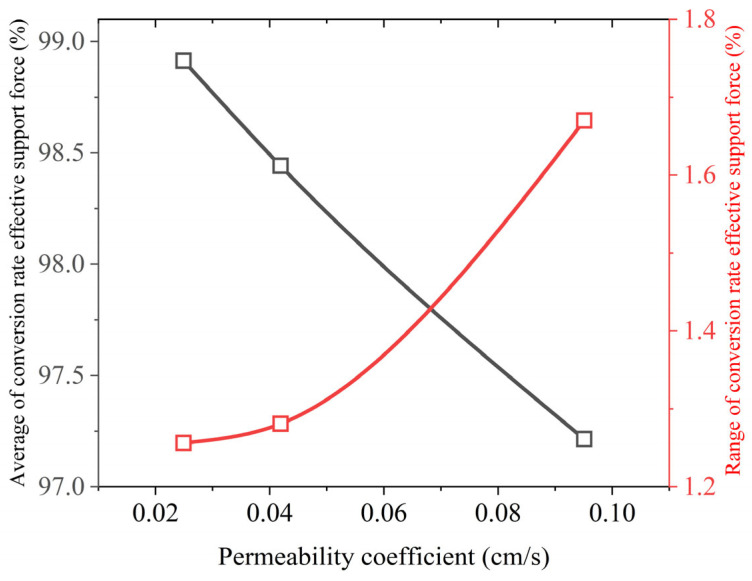
Relationship between the average, range, and stratum permeability coefficient.

Further analysis was conducted using the average value and range of the effective support force conversion rate curve from Figure 14b. These parameters indicate the magnitude and fluctuation degree of the support force at the excavation face, with the results shown in Figure 15. It can be observed that the average effective support force conversion rate has a linear relationship with the stratum permeability coefficient, while the range shows a nonlinear relationship. When the stratum permeability coefficient increases from 0.0419 cm/s to 0.0915 cm/s (corresponding to an increase in stratum particle size from 2–4 mm to 4–8 mm), there is a sudden increase in the range. This indicates that the increase in stratum permeability exacerbates the fluctuations in the effective support force conversion rate. It also explains the phenomenon of sudden and intense fluctuations in the pressure of the slurry chamber when the shield unexpectedly advances into a highly permeable stratum.

## 5. Discussion

In this chapter, the quantitative relationships between factors such as bentonite and CMC on slurry permeability are studied. By analyzing the effective support force conversion rate curves, the average value and range are used to represent the magnitude and stability of the slurry’s supporting capacity at the excavation face.

### 5.1. Effects of Different Factors on Effective Support Force Conversion Rate

#### 5.1.1. Influence of Slurry Materials on Effective Support Force Conversion Rate

The average and range of the effective support force conversion rate during the slurry filtration process are used as indicators to characterize the slurry’s supporting capacity and stability at the excavation face. These two metrics reflect the magnitude and fluctuation degree of the effective support force conversion rate, providing a comprehensive description of the characteristics of the effective support force conversion rate curve. The relationships between various factors and the aforementioned indicators are plotted as curves and fitted accordingly. The fine sand particle size is expressed as a mass of 0–0.5 mm particle size. Appendix A presents the raw data (average and range) of the effective support force conversion rate, along with corresponding fitting equations and R^2^ values quantifying relationships between influencing factors and both the mean values and range of this parameter.

**Figure 16:**
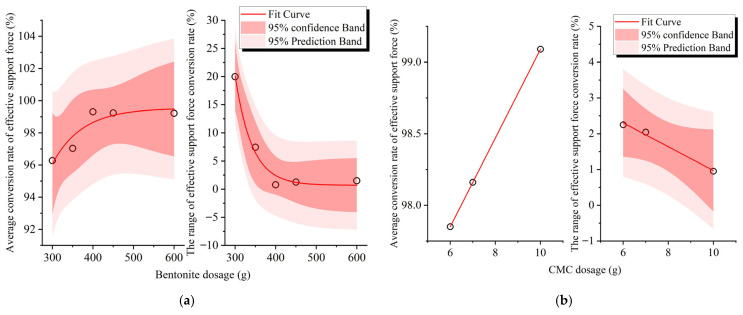

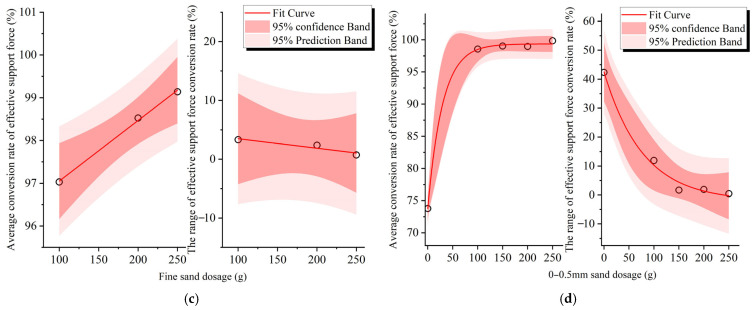
Influence of slurry materials on effective support force conversion rate: (**a**) bentonite content; (**b**) CMC content; (**c**) fine sand content; and (**d**) fine sand size.

The influence of slurry composition on the effective support force conversion rate is presented in Figure 16. A nonlinear relationship is observed between bentonite content and fine sand gradation with respect to both the mean and range of the conversion rate, whereas CMC content and fine sand quantity exhibit well-defined linear correlations with these metrics. The susceptibility of bentonite slurry to seawater-induced flocculation stems from cation infiltration: When seawater enters the slurry, abundant cations neutralize the negative surface charges of clay particles. This electrolyte intrusion compresses the diffuse double layer of clay colloids, diminishing interparticle repulsion forces. Consequently, the slurry system transitions from fine dispersion to coarse dispersion, forming flocculated structures that manifest macroscopically as rapid sedimentation and water separation [44].

SEM analysis of filter cakes confirms that seawater-exposed specimens develop more compact soil particle aggregates with enhanced intra-aggregate porosity and improved connectivity compared to seawater-free counterparts [44,45]. CMC counteracts these effects through hydrogen bonding with bentonite particles. Its molecular chains establish network structures that increase slurry viscosity, imparting cohesive density to flocculated structures. This mechanism preserves bentonite swelling capacity and maintains favorable rheological properties in seawater-contaminated slurries [2]. Fine sand primarily alters the particle size distribution of the slurry with minimal impact on its viscosity. As evident from Figure 16c, the quantity of fine sand exhibits well-defined linear correlations with both the mean value and range of the conversion rate. Collectively, these phenomena explain the nonlinear relationship between bentonite content and the mean/range of the effective support force conversion rate: bentonite differentially impacts slurry viscosity and particle size gradation, with increasing concentrations producing distinct effects on these parameters.

The nonlinear relationship between fine sand particle size and the indicators may be attributed to the fact that when the fine sand particle size is significantly different from the particle size that determines whether the slurry can form a filter cake at the excavation face [32], the impact of fine sand particle size on the effective support force conversion rate is substantial; conversely, if the particle sizes are similar, the effect is diminished.

#### 5.1.2. Effects of Tunneling Parameters and Stratum Permeability on Effective Support Force Conversion Rate

**Figure 17:**
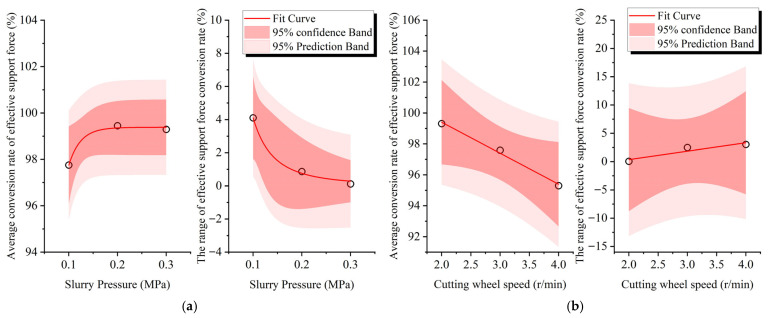

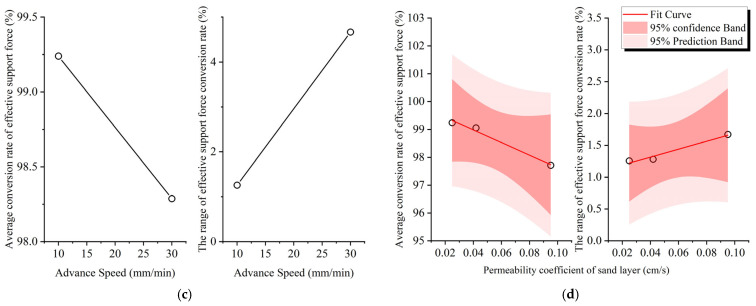
Effects of tunneling parameters and stratum permeability on effective support force conversion rate: (**a**) slurry pressure; (**b**) cutterhead rotation speed; (**c**) advance speed; and (**d**) stratum permeability coefficient.

The effects of tunneling parameters and stratum permeability on the effective support force conversion rate are illustrated in Figure 17. With the exception of the nonlinear relationship between slurry pressure and both the average value and range, all other relationships are linear. This indicates that enhancing the slurry’s supporting performance at the excavation face by increasing slurry pressure is most pronounced when the slurry pressure is relatively low.

### 5.2. Sensitivity Analysis of the Average Value and Range of Effective Support Force Conversion Rate

The data obtained in Section 5.1 were analyzed to determine the ratio of the average rate of change in the effective support force conversion rate (both the average value and range) to the average rate of change in each factor. This ratio serves as a parameter for assessing the relative importance of each factor. The calculation method is detailed in reference [27].

**Figure 18:**
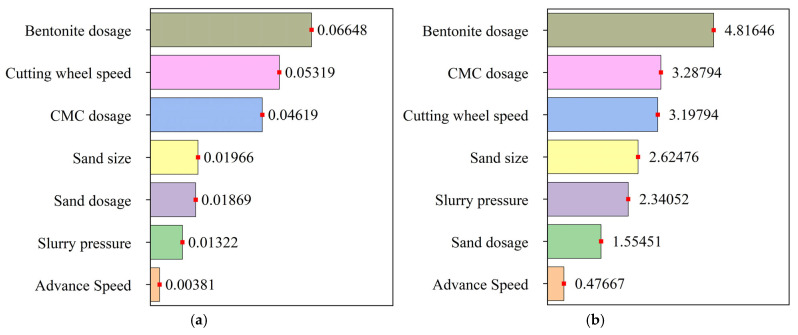
Results of sensitivity analysis on slurry supporting performance: (**a**) average value and (**b**) range.

The relative importance parameters of each factor were ranked, with the results shown in Figure 18. The ranking based on the relative importance of each factor to the average value of the effective support force conversion rate, from highest to lowest, is as follows: bentonite, cutterhead rotation speed, CMC, fine sand particle size, fine sand content, slurry pressure, and advance speed. For the relative importance to the range, the ranking from highest to lowest is bentonite, CMC, cutterhead rotation speed, fine sand particle size, slurry pressure, fine sand content, and advance speed.

By comparing the values of the relative importance parameters for each factor, it can be observed that the impact of the factors on the range of the effective support force conversion rate is greater than their impact on the average value. The support force at the excavation face is more susceptible to fluctuations [46]. The amounts of bentonite and CMC are the two material factors that have the greatest influence on both indicators, with bentonite having a more significant effect on the effective support force conversion rate than CMC, a finding that is supported by the research of Ye et al. [47]. The cutterhead rotation speed is the most influential tunneling parameter; compared to the penetration depth of the cutter, the cutter speed has a more pronounced effect on the conversion rate of slurry support pressure at the tunnel face [48]. The heightened sensitivity of the effective support force conversion rate to bentonite content compared to CMC is primarily driven by bentonite’s dual functionality: it simultaneously modifies both particle size distribution and viscosity in the slurry system. Elevating bentonite content increases particulate density within the slurry, thus enhancing pore-plugging capacity in geological formations and promoting filter cake development. Increased slurry viscosity substantially reduces seepage velocity, facilitating bentonite particle retention within excavation face pores. Higher viscosity slurry further enables transportation of finer particulates, inducing particle bridging phenomena along seepage pathways that effectively enhance formation pore sealing [34]. In engineering applications, when introducing viscosity-enhancing additives like CMC, concurrently incorporating clay or silt to optimize particle size distribution synergistically enhances additive efficacy.

## 6. Conclusions

This study investigates the filtration characteristics of bentonite slurry under the cutting influence of the shield cutterhead in high-permeability strata within a subsea environment using a model testing device. The effects of slurry materials, including bentonite, CMC, and fine sand, as well as tunneling parameters such as slurry pressure, advance speed, and cutterhead rotation speed, on the effective support force conversion rate at the excavation face during the slurry filtration process were examined. The results indicate that.

(1) Under cutterhead cutting conditions, the pore water pressure rises immediately after the start of the experiment and fluctuates as the test progresses, while the effective support force conversion rate drops immediately after the experiment begins and fluctuates within a certain range, unable to reach 100%.

(2) Adding bentonite, CMC, and fine sand to the slurry, reducing the particle size of fine sand, increasing slurry pressure, and decreasing cutterhead rotation speed and advance speed help improve the effective support force conversion rate and enhance its stability.

(3) The effective support force conversion rate’s average value and range are used to characterize the support effect and stability of the slurry at the excavation face during cutterhead cutting, and the influence of various factors on both metrics was studied. The relationships between the average value and range of the effective support force conversion rate with bentonite content and fine sand particle size are nonlinear. The stratum permeability coefficient and slurry pressure exhibit a nonlinear relationship with the range and a linear relationship with the average value, indicating marginal effects of these factors on the average value and range.

(4) A sensitivity analysis of the average value and range of the effective support force conversion rate was conducted, with the ranking of the effects on the average value of effective support force conversion rate from greatest to least being bentonite, cutterhead rotation speed, CMC, fine sand particle size, fine sand content, slurry pressure, and advance speed. For the range of the effective support force conversion rate, the ranking from greatest to least is bentonite, CMC, cutterhead rotation speed, fine sand particle size, slurry pressure, fine sand content, and advance speed.

(5) Based on the experimental results of this study, the following slurry formulation and tunneling parameter adjustments are proposed for slurry shield tunneling in submarine formations characterized by high salinity and high permeability: the bentonite-to-water ratio should exceed 40%, the carboxymethyl cellulose (CMC) dosage should be greater than 10 g per liter of water, and the fine sand should have an optimal particle size ratio of 0–0.5 mm to 0.5–1 mm = 2:3. The speed of the cutterhead should not exceed 3 r/min, and the forward speed should be kept below 10 mm/min. These conclusions, derived from laboratory-scale tests, require further verification through engineering applications.

## Figures and Tables

**Table 1:** Composition of seawater for testing.

Components	NaCl	MgCl_2_	Na_2_SO_4_	CaCl_2_	KCl	NaHCO_3_
Content (g/L)	24.53	5.20	4.09	1.16	0.69	0.20
Proportion (%)	68.4	14.5	11.4	3.2	2.0	0.6

**Table 2:** Strata material parameters.

Stratum Particle Size (mm)	Permeability Coefficient (cm/s)
1~2	2.49 × 10^−2^
2~4	4.19 × 10^−2^
4~8	9.51 × 10^−2^

**Table 3:** Slurry material experimental arrangement.

Test ID	Freshwater (g)	Seawater (g)	Bentonite (g)	Fine Sand (g)	CMC (g)	Fine Sand 0–0.5 mm (g)	Fine Sand 0.5–1 mm (g)
1	700	300	300	250	10	0	0
2	700	300	350	250	10	0	0
3	700	300	400	250	10	0	0
4	700	300	450	250	10	0	0
5	700	300	600	250	10	0	0
6	700	300	450	250	6	0	0
7	700	300	450	250	7	0	0
8	700	300	450	200	10	0	0
9	700	300	450	100	10	0	0
10	700	300	450	0	10	250	0
11	700	300	450	0	10	150	100
12	700	300	450	0	10	100	150
13	700	300	450	0	10	0	250

**Table 4:** Shield tunneling parameter experimental arrangement.

Test ID	Strata Particle Size (mm)	Slurry Pressure (MPa)	Cutterhead Rotation Speed (r/min)	Advance Speed (mm/min)
1	1–2	0.3	2	10
2	1–2	0.3	3	10
3	1–2	0.3	4	10
4	1–2	0.3	4	10
5	1–2	0.3	4	30
6	1–2	0.1	4	10
7	1–2	0.2	4	10
8	1–2	0.3	4	10
9	1–2	0.3	4	10
10	2–4	0.3	4	10
11	4–8	0.3	4	10

## Data Availability

The original contributions presented in this study are included in the article. Further inquiries can be directed to the corresponding author.

## Appendix A

Table A1 presents the raw data of the effective support force conversion rate (average and range). Table A2 and Table A3 summarize the corresponding fitting equations and R^2^ values quantifying relationships between influencing factors and both the mean values and range of this conversion rate.

**Table A1:** The raw data of the effective support force conversion rate.

Influence Factor	Parameter Value	Average Conversion Rate of Effective Support force	The Range of Effective Support Force Conversion Rate
Bentonite dosage (g)	300	96.271	19.982
	350	97.036	7.437
	400	99.31	0.779
	450	99.239	1.256
	600	99.217	1.49
CMC dosage (g)	6	97.85	2.248
	7	98.16	2.042
	10	99.09	0.953
Fine sand dosage (g)	250	99.138	0.715
	200	98.526	2.369
	100	97.031	3.317
0–0.5 mm sand dosage(g)	250	99.831	42.343
	200	98.953	1.871
	150	99.02	1.634
	100	98.548	11.909
	0	73.747	42.343
Slurry Pressure (MPa)	0.3	99.449	4.098
	0.2	99.449	0.858
	0.1	97.751	4.098
Cutting wheel speed (r/min)	4	99.308	3.016
	3	97.59	2.488
	2	99.308	0.037
Advance Speed (mm/min)	30	99.239	4.666
	30	98.287	4.666
	10	99.239	1.256
Permeability coefficient of sand layer (cm/s)	0.0249	99.239	1.256
	0.0419	99.053	1.281
	0.0951	97.707	1.67

**Table A2:** Regression equations between various influencing factors and the average effective support force conversion rate.

Influence Factor	Regression Equations	R^2^
Bentonite dosage	$y=99.54344-197.90599\times e^{-0.01349x}$	0.86621
CMC dosage	y=95.98947+0.31009x	1
Fine sand dosage	y=95.63295+0.01417x	0.99776
0–0.5 mm sand dosage	$y=99.35535-25.60777\times e^{-0.03379x}$	0.99921
Slurry pressure	$y=99.38171(1-e^{-41.1159x})$	0.99021
Cutting wheel speed	y=103.41607−2.00622x	0.99317
Advance speed	/	/
Permeability coefficient of sand layer	y=99.88905−22.6573x	0.98497

**Table A3:** Regression equations between various influencing factors and the range of effective support force conversion rate.

Influence Factor	Regression Equations	R^2^
Bentonite dosage	$y=0.69618+27812.96802\times e^{-0.02422x}$	0.98417
CMC dosage	y=4.2991−0.33283x	0.99122
Fine sand dosage	y=5.10834−0.01622x	0.88529
0–0.5 mm sand dosage	$y=-2.09823+44.5591\times e^{-0.01282x}$	0.99108
Slurry pressure	$y=0.01503\times x^{-2.4364}$	0.9957
Cutting wheel speed	y=−2.62063+1.48919x	0.87798
Advance speed	/	/
Permeability coefficient of sand layer	y=1.06592+6.23537x	0.96758

## References

[1] Wang X., Zhang L., Zhang Q., Liu R., Huang C. (2025). A stepwise calculation method for grouting penetration in rough rock fracture based on fracture segment division. Tunn. Undergr. Space Technol..

[2] Wang Z., Ding W., Zhu Z., Liu R., Wang C., Yu W., Wang Z. (2022). Experimental study on rheological behaviors of Na-bentonite slurries under seawater intrusion. Constr. Build. Mater..

[3] Holzhaeuser J., Hunt S.W., Mayer C. (2006). Global experience with soft ground and weak rock tunneling under very high groundwater heads. Proceedings of the North American Tunneling 2006: North American Tunneling 2006 Conference.

[4] Han X.R., Zhu W., Liu Q.W. (2008). Influence of slurry property on filter cake quality on working face of slurry shield. Rock Soil Mech..

[5] Min F.-L., Zhu W., Han X.-R., Zhong X.-C. The Effect of Clay Content on Filter-Cake Formation in Highly Permeable Gravel. Proceedings of the Geoshanghai International Conference.

[6] Min F., Zhu W., Han X. (2013). Filter cake formation for slurry shield tunneling in highly permeable sand. Tunn. Undergr. Space Technol. Inc. Trenchless Technol. Res..

[7] Wu D., Zhou S., Wen X. (2015). Laboratory test and application of filter cake formation in sand during slurry shield construction. Chin. J. Rock Mech. Eng..

[8] Xu T., Bezuijen A. (2019). Bentonite slurry infiltration into sand, filter cake formation under various conditions. Géotechnique.

[9] Bai Y. (2022). Study on Slurry Penetration and Stability of Slurry Shield Tunnel Face Based on Interaction of “Cutter-Soil”. Ph.D. Thesis.

[10] Mao J., Yuan D., Chen J., Yang P., Liu S. (2024). Study on the Characteristics of Slurry Support Efficiency on Excavation Face Induced by Shield Cutterhead Cutting in Sand Stratum. KSCE J. Civ. Eng..

[11] Zdenek Z., Britta S., Markus T. (2021). Investigations on transient support pressure transfer at the tunnel face during slurry shield drive part 1: Case A—Tool cutting depth exceeds shallow slurry penetration depth. Tunn. Undergr. Space Technol. Inc. Trenchless Technol. Res..

[12] Nomoto T., Imamura S., Hagiwara T., Kusakabe O., Fujii N. (1999). Shield Tunnel Construction in Centrifuge. J. Geotech. Geoenvironmental Eng..

[13] Liu X., Xiong F., Zhou X., Liu D., Chen Q., Zhang J., Han Y., Xu B., Deng Z., He C. (2022). Physical model test on the influence of the cutterhead opening ratio on slurry shield tunnelling in a cobble layer. Tunn. Undergr. Space Technol..

[14] Yang J., Liu C., Chen Q., Xie X. (2017). Performance of overlapped shield tunneling through an integrated physical model tests, numerical simulations and real-time field monitoring. Undergr. Space.

[15] Hu X., He C., Walton G., Fang Y., Dai G. (2020). Laboratory Model Test of EPB Shield Tunneling in a Cobble-Rich Soil. J. Geotech. Geoenvironmental Eng..

[16] Ma S., Duan Z., Huang Z., Liu Y., Shao Y. (2022). Study on the stability of shield tunnel face in clay and clay-gravel stratum through large-scale physical model tests with transparent soil. Tunn. Undergr. Space Technol..

[17] Hu X., Fang Y., Walton G., He C. (2023). Laboratory model test of slurry shield tunnelling in saturated sandy soil. Géotechnique.

[18] Shen X., Yuan D., Jin D., Chen X., Luo W., Peng Y., Duan K. (2025). Model test on cutterhead-soil interaction during shield tunneling and its theoretical model. Undergr. Space.

[19] Mao J., Yuan D., Jin D., Liu S. (2021). Influence of Cutting Tools on Filter Cake Formation during Slurry Shield Tunnelling. KSCE J. Civ. Eng..

[20] Kong X., Bai Y., Xu D. (2012). The Elastic Modulus of Slurry Membrane in Excavation Face for Slurry Shield. Chin. J. Undergr. Space Eng..

[21] Xisheng Y. (2017). Cake Filtration and Face Stability of Slurry Shield Tunnel. Ph.D. Thesis.

[22] Jin D., Yuan D. (2022). Face stability analysis of the slurry-type shield tunneling considering the dynamic filter cake. Rock Soil Mech..

[23] Wang Q., Xia Y., Huang S., Yang M., Zhang L., Sheng J., Xiao H. (2024). Numerical Investigation of the Impact of Tunneling Parameters on the Mud Discharge Performance of Atmospheric Cutterhead. Ksce J. Civ. Eng..

[24] Zizka Z., Schoesser B., Thewes M., Schanz T. (2019). Slurry Shield Tunneling: New Methodology for Simplified Prediction of Increased Pore Pressures Resulting from Slurry Infiltration at the Tunnel Face Under Cyclic Excavation Processes. Int. J. Civ. Eng..

[25] Mao J.H. (2021). Research on Laws of Dynamic Filter Cake Formation and Face Stability During Slurry Shield Tunnelling in Sand Stratum. Ph.D. Thesis.

[26] Qin S., Cheng Y., Zhou W.-H. (2023). State-of-the-art review on pressure infiltration behavior of bentonite slurry into saturated sand for TBM tunneling. Smart Constr. Sustain. Cities.

[27] Dong H., Wang D., Li Z., Zhang Q., Li Y., Zhang J., Zhang L., Zhang L. (2024). Experimental Study on Infiltration of Seawater Bentonite Slurry. Buildings.

[28] Lin Y., Fang Y., He C., Wang W. (2021). Experimental study on degree of match between slurry and ground based on particle retention rate. Tunn. Undergr. Space Technol..

[29] Chen K., Hong K., Jiao S. (2016). Shield Construction Technology.

[30] Broere W. (2002). Influence of excess pore pressures on the stability of the tunnel face. Geotechnical Aspects of Underground Construction in Soft Ground.

[31] Broere W., Van Tol A.F. (2000). Influence of infiltration and groundwater flow on tunnel face stability. Geotech. Asp. Undergr. Constr. Soft Ground.

[32] Broere W. (2001). Tunnel Face Stability and New CPT Applications. Ph.D. Thesis.

[33] Wei D.W., Zhu W., Min F.L. (2014). Experimental study of forming time of filter cake and conversion rate of slurry pressure in slurry shield in sand stratum. Rock Soil Mech..

[34] Chen Y., Cai Y., Ye W., Cui Y., Chen B. (2021). Progresses in researches on adsorption and migration properties of bentonite colloids and their co-migration with nuclide in repository. Chin. J. Geotech. Eng..

[35] Ding H. (2019). Seepage Law and Prediction of Water Inflow in Surrounding Rock of Weathering Trough in Xiamen Subsea Tunnel. Master’s Thesis.

[36] Zhang H., Shan X. (2022). Quantitative Measurement Research on Slurry Blending and Pulping Engineering of Slurry Balance Shield. Railw. Eng. Technol. Econ..

[37] Ding X. (2023). Research on Key Construetion Technology of Slurry Shield Crossing Shiziyang Fault Fracture Zone. Eng. Technol. Res..

[38] Yin Z., Zhang Q., Zhang X., Li X., Zheng D. (2024). Effect of seawater on bentonite slurry and its infiltration behavior into saturated sand for tunnelling. Bull. Eng. Geol. Environ..

[39] Yang Y., Yuan D., Jin D., Yi Y. (2024). Development and experiment study on seawater-based slurry for subsea tunnels in slurry shield tunneling. Constr. Build. Mater..

[40] Yang F. (2024). Research on the Parameter Law and Optimization of Shield Tunneling in Hard Rock Composite Strata in Sea Area. Railw. Constr. Technol..

[41] Huang Q. (2010). Research on Interaction with Soil of TBM Cutting-Wheel Tools and Their Type Selection Design in Gravel Stratum. Ph.D. Thesis.

[42] Bai Y., Jiang B., Yang L., Liu Y., Zheng H., Li Y. (2022). Experimental Study on the Characteristics and Formation Mechanism of Dynamic Filter Cake for Slurry Shield Tunneling. Minerals.

[43] Yang Y., Jin D., Yuan D., Yao Z. (2024). Spatial distribution of excess pore water pressure and slurry infiltration zone in slurry shield tunneling. Tunn. Undergr. Space Technol..

[44] Min F., Du J., Zhang N., Chen X., Lv H., Liu L., Yu C. (2019). Experimental study on property change of slurry and filter cake of slurry shield under seawater intrusion. Tunn. Undergr. Space Technol..

[45] Qin S., Zhou W.H., Xu T. (2023). Effects of seawater on the infiltration behavior of bentonite slurry into sand. Constr. Build. Mater..

[46] Wei G., Zhu Y., Yin X. (2021). Research progress of slurry penetration test and filter cake forming law of slurry shield. Low Temp. Archit. Technol..

[47] Ye W., Fu L., Zhou S. (2022). Influence of Shield Slurry Property on Filter Cake Quality in Sand Stratum. Advances in Transportation Geotechnics IV, Proceedings of the 4th International Conference on Transportation Geotechnics, Chicago, IL, USA, 23–26 May 2021.

[48] Liu K., Yang R., Wang S., Zhao W., Qi P. (2024). Slurry Support Mechanism for Shield Tunneling Considering Slurry Infiltration and Cutter-Disk Cutting Effect. Int. J. Geomech..

